# Evaluation of different airway tests to determine difficult intubation in apparently normal adult patients: undergoing surgical procedures

**DOI:** 10.1186/s13037-020-00263-5

**Published:** 2020-11-22

**Authors:** Khaled El-Radaideh, Ehab Dheeb, Hamzeh Shbool, Saif Garaibeh, Adel Bataineh, Wail Khraise, Basil EL-Radaideh

**Affiliations:** 1 Department of Anesthesiology and Intensive Care, Faculty of Medicine, Jordan University of Science and Technology, P.O. Box 953, Irbid, 21110 Jordan; 2 Intern in the department of general surgery, Faculty of Medicine, Jordan University of Science and Technology, P.O. Box 953, Irbid, 21110 Jordan

**Keywords:** Airway management, Cormack-Lehane, Difficult intubation, Laryngoscopy, Mandibular protrusion, Modified Mallampati test, Sternomental distance, Thyromental distance

## Abstract

**Background:**

Inadequate maintenance of a patient’s airway represents a major cause of anesthesia-related morbidity and mortality. This study was designed to evaluate common preoperative clinical tests to determine the risk of difficult endotracheal intubation in apparent “normal” adult patients undergoing surgical procedures.

**Methods:**

A prospective observational cohort study was performed on 160 consecutive adult patients undergoing surgical procedures at an academic medical center in Jordan from 20 May 2019 until 11 February 2020.

Preoperative assessment of airway risk stratification was performed by the following clinical tests: the mandible protrusion test (MPT), thyromental (TMD) and sternomental (SMD) distances, inter-incisor gap (IIG), and the modified Mallampati tests with tongue protrusion (MMT-TP) and without tongue protrusion (MMT-NTP). Grade C on the MPT, TMD ≤ 6 cm, SMD ≤ 12 cm, and MMT grades III and IV were considered to be predictors of difficult endotracheal intubations. A modified Cormack-Lehane grading (MCLG) of laryngoscopic views with backward, upward, and right-sided pressure on the thyroid and cricoid cartilages (BURP) maneuver was also documented, with grades 2B, 3, and 4 considered to be difficult airways for intubation.

**Results:**

Fifteen patients (9.4%) were classified as MCLG 2B, 3, and 4, with age significantly associated with the MCLG grade (*P* = 0.028). The sensitivity and Youden’s index of MMT-TP were found to be the lowest (40% and 0.29, respectively). The MPT was the most accurate and specific test (90.63 and 95.17%, respectively), with the highest PPV (50%), Youden’s index (0.42), and area under the curve (AUC) (0.781). Bivariant analysis of MPT and the *t*-test of the mean TMDs and SMDs revealed significant associations between these airway tests and the difficulty of intubation (*P* values: < 0.001, 0.02, < 0.01, respectively).

**Conclusion:**

The MPT, with its highest accuracy, specificity, positive predictive value, and good sensitivity may be used as a routine screening test for preoperative prediction of difficult endotracheal intubations.

## Introduction

The prevalence of difficult laryngoscopic intubations is reported to range from 1.5 to 20% [1–3].

Unanticipated difficult intubations remain a major concern for anesthesiologists due to the potentially serious consequences of failed endotracheal intubations [4]. The identification of patients with difficult airways is crucial during preoperative evaluations [5]. A variety of tests are used to evaluate for a potentially difficult intubation in advance of the procedure [6, 7]. It is not clear; however, which test has the best predictive ability.

Therefore, we conducted this prospective study to evaluate the accuracies of the mandibular protrusion test (MPT), thyromental distance (TMD), sternomental distance (SMD), inter-incisor gap (IIG), and the modified Mallampati test (MMP) for prediction of difficult intubations relative to the modified Cormack-Lehane grading (MCLG) with backward, upward, and right-sided pressure on the thyroid and cricoid cartilages (BURP) maneuver for difficult laryngoscopic intubations. The main goal of the study was to determine which airway assessment test and/or combination of tests was best at predicting difficult intubations.

## Methods

After the institutional research Ethics Committee approval of this observational, prospective study (IRB approval number 20190210), we obtained written informed consent from all patients.

A prospective observational cohort study was performed on 160 consecutive adult patients with American Society of Anesthesia (ASA) class I, II and III who required endotracheal intubation for elective surgical procedures at King Abdullah university hospital in Irbid, Jordan from 20 May 2019 until 11 February 2020.

Patients were excluded from the study if they met any of the following criteria: 1) age < 18 years; 2) pregnancy; and patients scheduled for cesarean section; 3) increased risk of pulmonary aspiration; 4) body mass index of 35 kg/m2 or greater; or 5) inability to communicate (e.g. confusion, poor hearing, or language barrier); 6) abnormal patients (patients with a history of difficult intubation or physical signs of abnormal anatomy).

Patients were premedicated with 5 mg diazepam orally on the evening before surgery.

Upon arrival at the anesthetic room, all patients received an intravenous catheter. Routine monitoring included electrocardiography (ECG), pulse oximetry, noninvasive blood pressure, and end-expiratory gas analysis. After preoxygenation, anesthesia was induced in all patients with 2 μg/kg of fentanyl and 2 to 3 mg/kg of propofol. Neuromuscular blockade was achieved with 0.5 mg/kg of atracurium besilate. Patient lungs were hand-ventilated via facemask with 1% sevoflurane and 100% oxygen till the neuromuscular block was completed.

Direct laryngoscopy was performed, and a MCLG of laryngoscopic views with the BURP maneuver was documented. This 5-grade scoring system involves the subdivision of the original grade 2 into 2A (partial view of glottis is visible) and 2B (only the arytenoids are visible) [7, 8]. Grades 2B, 3, and 4 were considered to be difficult intubations.

Controlled ventilation through an endotracheal tube was maintained with 40% O2 in air and 1 to 1.2 minimum alveolar concentration of sevoflurane. Mechanical ventilation was set to maintain an end-tidal carbon dioxide (CO2) between 32 and 40 mmHg. At the end of surgery, residual neuromuscular blockade was reversed with neostigmine and atropine. The sevoflurane was discontinued 3 to 5 min before completion of the surgical procedure.

### Measurements

One day prior to surgery, measurements were obtained by anesthesiologists not involved in endotracheal intubations of the study participants. Data was documented in an allocated data sheet.

We documented the grades of the MMT, according to the Samsoon and Young [9] airway classification. This measurement was performed in a sitting posture with a neutral head position and the tongue maximally protruded from the mouth without phonation (MMT-TP). Results were categorized into four classes, in which class III (only the soft palate could be seen) and class IV (the soft palate was not visible) were considered to be predictors for difficult endotracheal intubations. Patients with class I (soft palate, fauces, uvula, and pillars could be seen) and class II (soft palate, fauces, and uvula could be seen) were predicted to have easier intubations.

The MPT was assessed based on the classification system of UlHaq et al. [10], and is described as follows: class A = lower incisors can be protruded anterior to the upper incisors; class B = lower incisors can be brought edge-to-edge with the upper incisors; and class C = lower incisors cannot be brought edge-to-edge with the upper incisors. Class C was considered to be predictive of a difficult endotracheal intubation.

**Fig. 1:**
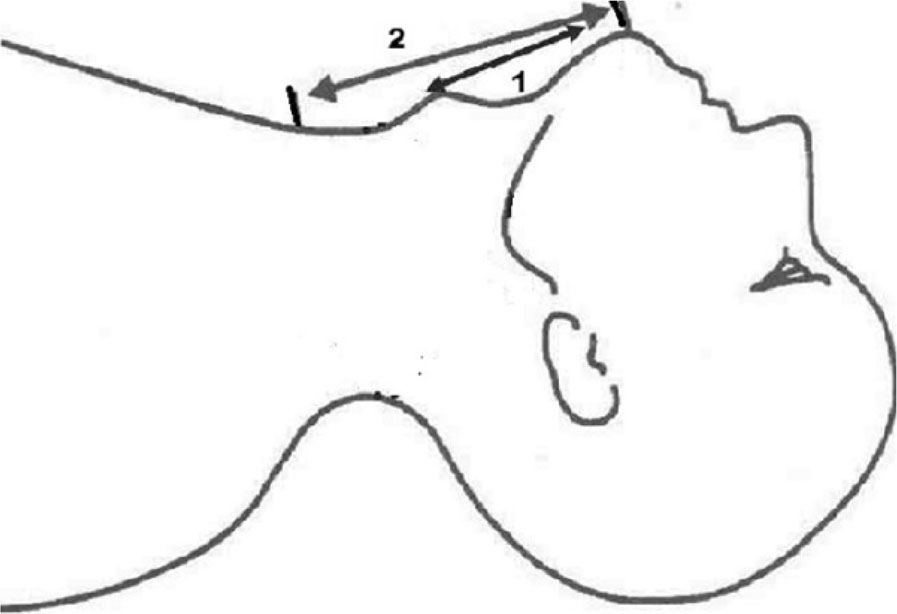
Measurement of the thyromental (1) and sternomental (2) distances

The thyromental distance (TMD) was measured from the tip of thyroid cartilage to the tip of mentum. A difficult intubation was predicted in patients with a thyromental distance of 6 cm or less. The sternomental distance (SMD) was measured from the sternal notch to the tip of insight of the mentum. TMD and SMD were measured with the neck fully extended and the mouth closed, using a ruler approximated to the nearest 0.5 cm [Fig. 1]. TMDs of ≤6 cm and SMDs of ≤12 cm were considered to be predictors for difficult visualizations of the larynx and difficult endotracheal intubations.

On the day of surgery, the inter-incisor gap (IIG) and the modified Mallampati test without phonation (MMT-NTP) were performed in the supine position and documented. Measurements of IIG were obtained at the midline between the upper and lower incisors while the patient was in the supine position, with maximum mouth opening and a neutral head position [11]. The MMT-NTP was performed in the supine posture with a neutral head position, without tongue protrusion and without phonation. Its classification schema was the same as that of the MMT-TP.

### Statistical analysis

The sample size was calculated with a precision error of 5% and type I error of 5%. We assumed an incidence of 11% for difficult laryngoscopies, based on a previously published study [12]. According to Eq. 1, the desired number of patients was 151. In anticipation of losses, and for more adequate control of potential confounding effects of variables, we enrolled 160 patients in this study.

1 $$ n={\left(Z1-\alpha /2\right)}^2\ast \mathrm{P}\ \left(1-\mathrm{P}\right)/{\mathrm{E}}^2 $$

*n* = number of patients in the sample. Z*1-α*/2 = 1.96. P = expected proportion in population based on previous study. E = precision error of 5%.

Continuous demographic data and continuous predictors of difficult intubation were presented as means ± SD. Number of patients was analyzed with a *t*-test. Pearson chi-square tests were used for categorical variables. Logistic regression analysis was performed to determine the predictors for difficult intubation in patients. Data analyses were performed using the Statistical Package for Social Sciences version 18 (SPSS Inc., USA). Statistical significance was considered as a *P*-value of 0.05 or less.

## Results

A total of 160 consecutive adult patients with age ≥ 18 years who were scheduled for elective surgical procedures requiring general anesthesia with endotracheal intubation were enrolled in this study. Patient characteristics are shown in Table 1.

**Table 1:** Patient characteristics and duration of anesthesia

Variable	Females (*n* = 92)	Males (*n* = 68)	*P*-Value	Total (*n* = 160)
Age (years)	40.98 ± 13.13	36.01 ± 14. 02	0.024	38.88 ± 13.70
Weight (kg)	75.69 ± 13.15	81.15 ± 15.13	0.019	78.01 ± 14.23
Height (cm)	163.52 ± 6.33	175.25 ± 7.22	< 0.001	168.50 ± 8.87
BMI (kg/m2)	28.26 ± 4.30	26.35 ± 4.25	0.006	27.44 ± 4.37
ASA				
I	46	25		71
II	40	39		79
III	6	4		10
Duration of Anesthesia (min.)	95.75 ± 48.24	104.43 ± 51.40	> 0.05	99.44 ± 49.79

Data are presented as mean ± standard deviation, or number. *BMI* body mass index, *ASA* American Society of Anesthesia physical status

Patient weight ranged from 45 to 112 kg, with 11 patients weighing from 100 to 105 kg and three patients weighing from 106 to 112 kg.

Mallampati scores III and IV were considered as predictors for difficult intubations. Among 22 patients with Mallampati scores of III or IV using the MMT-TP and 41 patients with Mallampati scores of III or IV using the MMT-NTP, only 6 and 9 patients, respectively, were truly difficult to intubate. Among 14 patients with a class C MPT, only seven patients were truly difficult to intubate (Table 2).

**Table 2:** Airway test parameters

	Females (*n* = 92)	Males (*n* = 68)	All (*n* = 160)
MMT-TP			
I and II	79 (85.9%)	59 (86.8%)	138 (86.25%)
III and IV	13 (14.1%)	9 (13.2%)	22 (13.75%)
MMT-NTP			
I and II	68 (73.9%)	51 (75.0%)	119 (74.4%)
III and IV	24 (26.1%)	17 (25.0%)	41 (25.6%)
Mandibular protrusion test			
Grades A and B	81 (88.0%)	65 (95.6%)	146 (91.25%)
Grade C	11 (12.0%)	3 (4.4%)	14 (8.75%)
Inter-incisor gap	4.57 ± 0.78	4.64 ± 0.67	4.60 ± 0.73
Thyromental distance, cm	6.98 ± 1.55	7.27 ± 1.39	7.1 ± 1.49
Sternomental distance, cm	14.32 ± 2.72	15.01 ± 2.82	14.61 ± 2.77

Data are given as mean ± standard deviation, or numbers (percentages). MMT-TP, modified Mallampati test with tongue protrusion; MMT-NTP, modified Mallampati test without tongue protrusion. Grade A: lower incisors can be brought anterior to the upper incisors. Grade B: lower incisors can only be protruded edge-to-edge with upper incisors. Grade C: lower incisors cannot be protruded edge-to-edge with upper incisors

Fifteen patients (9.38%) were found to have airways that were difficult to intubate during laryngoscopy. This incidence of difficult intubations represents the sum of the true-positive (TP) and false-negative (FN) cases.

This study had no occurrences of failed intubations. The tracheas of 11 patients were intubated using a standard endotracheal tube introducer (a so-called gum elastic bougie). Fiberoptic intubation was necessary in the remaining four patients.

MMT-NTP had the highest sensitivity (60%) and the lowest positive predictive value (PPV) (21.95%) and specificity (77.93%). The sensitivity and the Youden’s index of MMT-TP were found to be the lowest (40% and 0.29, respectively). The MPT was the most accurate and specific test (90.63 and 95.17%, respectively). This test also had the highest PPV (50%), Youden’s index (0.42), and area under the curve (AUC) (0.781).

Values for TPs, FNs, true negatives (TNs), false positives (FPs), accuracy ([TP + TN]/[TP + TN + FP + FN]), sensitivity (TP/ [TP + FN]), specificity (TN/[TN + FP]), PPV (TP/[TP + FP]), negative predictive value (NPV) (TN/[TN + FN]), and Youden’s index for MMT-TP, MMT-NTP, MPT, TMD, and SMD are shown in Table 3.

**Table 3:** Validity of airway assessment tests for predicting difficult intubations. Area under receiver operating characteristic curve of various airway assessment parameters

	MMT-TP	MMT-NTP	MPT	TMD	SMD
TP	6	9	7	7	8
FP	16	32	7	16	20
TN	129	113	138	129	125
FN	9	6	8	8	7
Accuracy %	84.38	76.25	90.63	85	83.1.3
Sensitivity %	40	60	46.67	46.67	53.33
Specificity %	88.97	77.93	95.17	88.97	86.20
PPV %	27.27	21.95	50	30.43	28.6
NPV %	93.48	94.96	94.52	94.16	94.7
Youden’s index	0.29	0.38	0.42	0.36	0.40
AUC	0.559	0.625	0.781	0.343	0.310
95% CI	0.407–0.710	0.493–0.757	0.655–0.907	0.198–0.488	0.170–0.451

*MMT-TP* modified Mallampati test with tongue protrusion, *MMT-NTP* modified Mallampati test without tongue protrusion, *MPT* mandibular protrusion test, *TMD* thyromental distance, *SMD* sternomental distance, *TP* true positive, *FP* false positive, *TN* true negative, *FN* false negative, *PPV* positive predictive value, *NPV* negative predictive value, *AUC* area under curve, and *CI* confidence interval

**Fig. 2:**
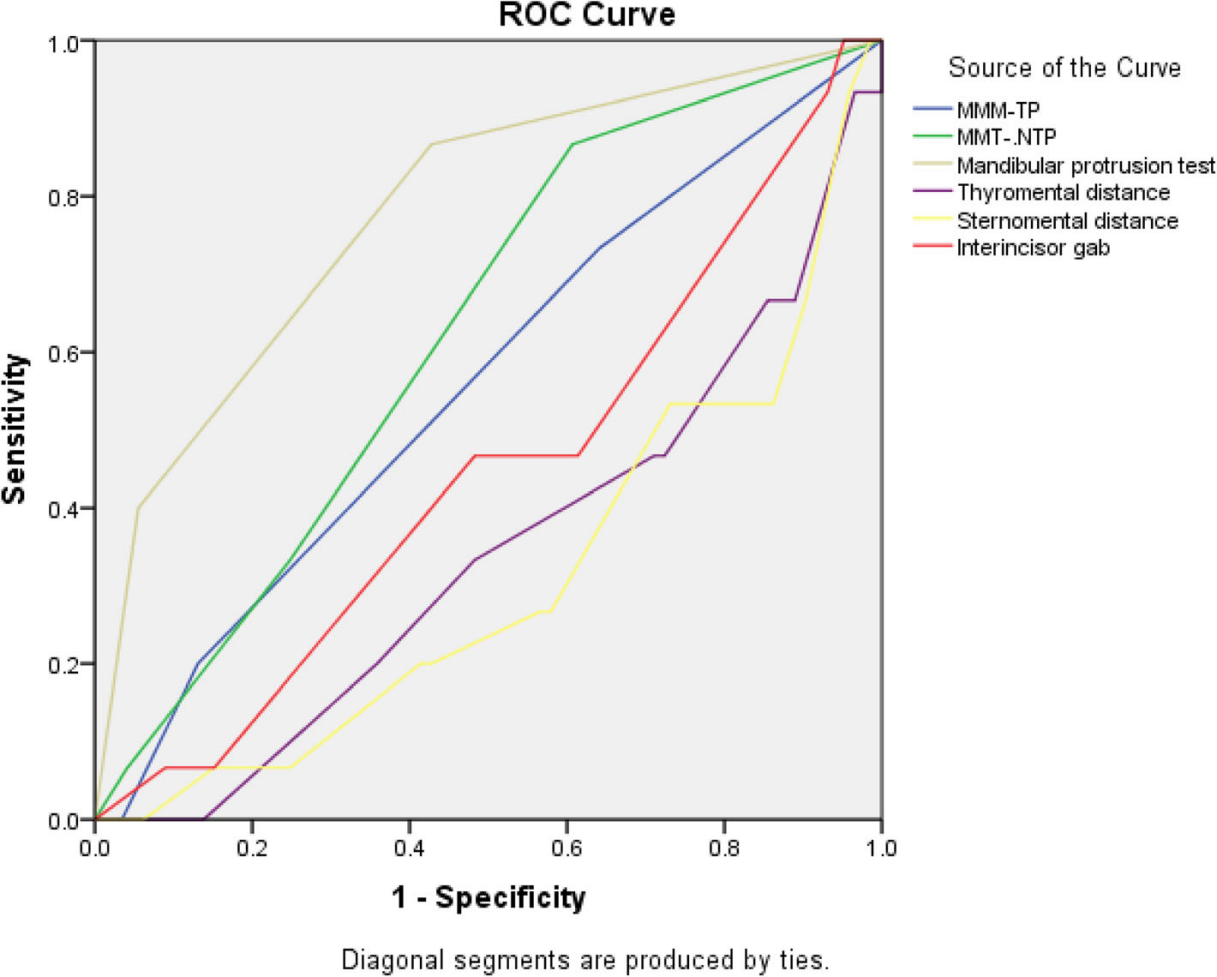
Receiver operating characteristic curve analysis of the airway tests. MMT-TP, modified Mallampati test with tongue protrusion; MMT-NTP, modified Mallampati test without tongue protrusion

Receiver operating characteristic curves (ROC) and AUC were used to identify the predictive abilities of the clinical tests (Fig. 2). The highest AUC was for MPT and the lowest AUC was for SMD (0.781 and 0.310, respectively) (Table 3).

Using the *t*-test, continuous variables, including weight, height, and BMI, were not significantly associated with the MCLG with BURP *(P-*values of 0.674, 0.387, and 0.263, respectively). Patient age, however, was significantly associated with the MCLG with BURP (*P* = 0.028). Associations between different airway tests and the difficulty of intubations obtained by bivariant analysis for preoperative variables are shown in Table 4.

**Table 4:** Association of airway assessment tests with modified Cormack-Lehane grading with BURP

	Cormack-Lehane grading with BURP		*P*-Value
	Grades 1 and 2A (Easy, *n* = 145)	Grades 2B and 3 (Difficult, *n* = 15)	
MMT with tongue protrusion			0.437
Mallampati I and II	129	9	
Mallampati III and IV	16	6	
MMT without tongue protrusion			0.536
Mallampati I and II	113	6	
Mallampati III and IV	32	9	
Mandibular protrusion test			< 0.001
Grade A and B	138	8	
Grade C	7	7	
Inter-incisor gap (cm)	4.61 ± 0.74	4.5 ± 0.68	0.569 t
Thyromental distance (cm)	7.19 ± 1.47	8.00 ± 1.47	0.021 t
Sternomental distance (cm)	24.00 ± 14.80	12.80 ± 2.80	0.007 t

Data are presented as mean ± standard deviation, or number. BURP, backward, upward, and right-sided pressure on the thyroid and cricoid cartilages; MMT, modified Mallampati test; t, Student’s *t*-test

The combination of various airway assessment tests are shown in Table 5.

**Table 5:** Validity of airway assessment test combinations in predicting difficult intubations

	Accuracy %	Sensitivity %	Specificity %	PPV %	NPV %	Youden’s index
MMT-TP + MMT-NTP	80.31	50	83.44	23.81	94.16	0.33
MMT-TP + TMD	84.69	43.33	88.96	28.89	93.82	0.32
MMT-TP + SMD	82.5	46.67	86.21	25.92	93.98	0.33
MMT-TP + MPT	87.5	43.33	92.07	36.11	94.01	0.35
MMT-NTP + TMD	80.62	53.33	83.45	25	94.53	0.37
MMT-NTP + SMD	78.43	56.67	80.69	23.29	94.74	0.37
MPT + MMT-NTP	83.44	53.33	86.55	29.09	94.72	0.4
MPT + SMD	85.63	50	89.31	32.61	94.53	0.39
MPT + TMD	87.81	46.67	92.061	37.84	94.35	0.39
TMD + SMD	82.3	46.87	86.21	27.27	93.63	0.33

*MMT-TP* modified Mallampati test with tongue protrusionm, *MMT – NTP* modified Mallampati test without tongue protrusion, *MPT* mandibular protrusion test, *TMD* thyromental distance, *SMD* sternomental distance, *PPV* positive predictive value, *NPV* negative predictive value

The categorical variables of sex and ASA were not strongly associated with the difficulty of endotracheal intubations.

## Discussion

Unanticipated difficult endotracheal intubations are the most common cause of anesthesia-related morbidity and mortality [13, 14], and are a major source of concern for anesthesiologists. As a result, it is important to identify a clinical test that is quick and easy to perform during a preoperative evaluation in order to accurately predict potentially difficult endotracheal intubations with high sensitivity and specificity [15].

In a study by Prakash and Ravi, no test could be identified that reliably predicted the majority of difficult intubations with a low false-positive rate [16]. The incidence of difficult intubations in the present study was identified to be 9.38%. In accordance with our results, Iohom et al. [17] reported an incidence of difficult intubations of 9%. Domi [18] encountered a difficult endotracheal intubation in 40 out of 426 patients (9.38%). The incidence of difficult intubations varied in other studies from between 3.4 to 23% [19, 20]. Differences in reported incidences may have been due to the diversity of definitions for difficult intubations [2, 7] or differences in anatomical structures of the patients [4, 21]. The amount of clinical experience of the anesthetists who are performing the endotracheal intubations may also have played an important role in previous assessments of the difficulty of an endotracheal intubation.

The incidence of difficult laryngoscopies may be improved by use of the BURP maneuver. Even in pediatric patients and with usage of a glidescope, Hirabayashi et al. [22] found that the BURP maneuver provided better glottis views. In contrast, Lee et al. [23] used the Clarus Video System and found that the BURP maneuver actually worsened the laryngeal view compared with the conventional maneuver. They also found that the MCLG was improved with the modified jaw thrust maneuver compared with the conventional maneuver.

For predicting difficult intubations, the MPT is a well-established and relatively simple grading system [10]. Savva [24] reported that protrusion of the mandible was too insensitive for routine use, with a sensitivity of 29.4%, specificity of 85%, and PPV of 9.1%. In that study, no patients were classified as grade C.

On the other hand, Yildiz et al. [25] found the incidences of difficult intubations in patients with mandibular protrusion grades of B or C were significantly lower than in patients with MMT scores of III or IV, with a lower sensitivity than observed in our study (31% vs. 46.67%, respectively). That study identified a higher number of patients with grade C than we did (32 vs. 14, respectively).

When comparing MPT-related results from a study by UlHaq et al. with our results, a higher sensitivity (46.67% vs. 95.88%, respectively), PPV (70.56% vs. 50%, respectively), and AUC (0.781 vs. 0.922, respectively) were found by UlHaq et al. [10]. The reported specificity and accuracy, however, were similar to the values identified in our study. The differences in the reported findings may have been attributable to inter-observer variability, inability of some patients to protrude the lower incisors anterior to the upper incisors, the diversity of definitions of difficult intubation, and the use of different patient populations [24, 26, 27].

Previous studies reported various cut-off points for TMD that could predict a difficult airway for intubation. Honarmand et al. [28] reported a TMD of ≤7.1 cm as a cut-off value for a difficult intubation. Badheka et al. [29] suggested 6 cm as the cut-off point for difficult intubations, and reported a sensitivity, specificity, PPV, and NPV of 70.59, 68.63, 84, and 50%, respectively, using that value. In our study we considered a TMD of ≤6 cm as a predictor for difficult endotracheal intubations. We found a TMD sensitivity, specificity, PPV, NPV, and Youden’s index of 46.67, 88.97, 30.43, 94.16%, and 0.36, respectively. The AUC for TMD was 0.343 (CI 0.198–0.488). Differences in TMD-related findings could be explained by factors that might influence the measurement of TMD, including limitation of head extension, shortness and depth of the mandible, and the height of the larynx [28]. As a result, some authors have doubted the reliability of TMD as an isolated predictive test for difficult laryngoscopies and intubations [30, 31]. On the other hand, Benumof [32] found that both a large and small TMDs could predict difficult intubations.

In our study, the sensitivity, specificity, and Youden’s index of 40, 88.9%, and 0.29, respectively (PPV = 27.3, NPV = 93.5), for MMT-TP supports findings of Shiga et al. [33], whose meta-analysis was comprised of 41,193 patients. That meta-analysis identified an overall sensitivity and specificity for MMT-TP of 49 and 86%, respectively. Similar results were reported by Iohom et al. [17]. Our results differed from some studies that have reported a higher sensitivity [34–36], and from those of Hashim et al. [27], who evaluated five airway tests in 60 patients of both genders, and found a 23% sensitivity, 68% specificity, 58% accuracy, and 16% PPV of the Mallampati test, which were smaller in comparison to our study. The wide variations in the reported sensitivities and specificities of the MMT may be due to the considerable inter-observer variability found during this assessment, which related to the performance of the test with or without phonation, patient cooperation, or patient position [16, 37].

In the present study, we performed the MMT-NTP in the supine position before the induction of anesthesia. We found an increase in the sensitivity (60%) using this technique; however, the PPV, specificity, and AUC were reduced in comparison to the results obtained using MMT-TP in the sitting position. On the other hand, the number of false-positives for MMT-NTP in the supine position was two times higher than those of MMT-TP in the sitting position (32 vs. 16, respectively). Contrary to our findings, Hanouz et al. [38] reported that supine performance of the MMT-TP for predicting difficult endotracheal intubation was superior to performance in the sitting position. Bindra et al. [39], however, found no significant changes in the diagnostic performance of the MMT-TP in the sitting or the supine positions. Khan et al. [35] demonstrated that the Mallampati test correctly depicts difficult intubations when the test is performed without phonation.

SMD is anatomically easy to measure and is commonly used in clinical practice [40].

Previous studies have reported different cut-off points for SMD, with consistent values ranging from 12.5 to 13.5 cm [3, 6, 29]. In the present study, SMD values of ≤12 cm were considered to be predictors of difficult endotracheal intubations. In our study, SMD sensitivity was found to be 53.3%, specificity was 86.2%, PPV was 28.6%, NPV was 94.7%, and accuracy was 83.1%. These findings are consistent with the results of Palczynski et al. [40], who found a sensitivity of 60% and a PPV of 19% for SMD. A poor sensitivity and PPV for this test (8.3 and 3.4%, respectively) were observed by Khatiwada et al. [41] and, in a study by Shobha et al. [42], SMD sensitivity was found to be 3.3% and PPV was 6.25%.

Although repeatedly reported to be a good measure of head extension, previous studies have reported that the SMD has limited clinical value and fails to adequately and solely predict difficult intubations [33, 41, 42].

This study had several limitations, including its exclusion of pregnant women, obese patients with a BMI of ≥35 kg/m2, and emergency cases. Also, TMD and SMD were measured by different persons. Finally, we did not assess neck mobility or neck circumference, which might also be important factors in predicting difficult laryngoscopies.

In conclusion, we found an incidence of difficult intubations of 9.38%, with significant increases noted with increasing age. Ideally, any clinical test that is used for prediction of these difficult airways should be quick, simple, convenient, and practical. Unfortunately, there is still no individual test, or combination of tests, with 100% sensitivity (i.e., no false negatives) and 100% specificity (i.e., no false positives). While the Mallampati score is an established method for predicting difficult intubations, its relatively low sensitivity and specificity limit the practical value of the test.

## Conclusions

The mandibular protrusion test (MPT), with its high accuracy, specificity, positive predictive value, and good sensitivity, may be used as a routine screening test for preoperative predictions of difficult endotracheal intubations. The combination of MMT and MPT with TMD or SMD could be beneficial in daily medical practice to predict the difficulty of larynx visualizations and the subsequent difficulty of intubations.

## Data Availability

The datasets used during the current study are available from the corresponding author, Khaled EL-Radaideh, on reasonable request.

## Abbreviations

AUC: Area under the curve
BURP: Backward, upward, and right-sided pressure on the thyroid and cricoid cartilages
FN: False negative
FP: False positive
IIG: Inter-incisor gap
MCLG: Modified Cormack-Lehane grading
MMT-NTP: Modified Mallampati tests without tongue protrusion
MMT-TP: Modified Mallampati tests with tongue protrusion
MPT: Mandible protrusion test
NPV: Negative predictive value
PPV: Positive predictive value
SMD: Sternomental distance
TMD: Thyromental distance
TN: True negative
TP: True positive
